# The Patterns and Environmental Factors of Diversity, Co-Occurrence Networks, and Assembly Processes of Protistan Communities in Bulk Soils of Forests

**DOI:** 10.3390/microorganisms13061249

**Published:** 2025-05-28

**Authors:** Bing Yang, Lin Wu, Zhisong Yang, Zhihe Zhang, Wanju Feng, Weichao Zheng, Chi Xu

**Affiliations:** 1 Sichuan Academy of Giant Panda, Chengdu 610081, China; imvinin@163.com (L.W.); yangzhisong@126.com (Z.Y.); 19828320401@163.com (W.F.); zheng_wei@nwafu.edu.cn (W.Z.); xuchi1979@163.com (C.X.); 2 Giant Panda National Park Chengdu Administration, Chengdu 610096, China; zhangzhh@163.com

**Keywords:** beta net relatedness index, forest type, functional groups, Illumina sequencing, neutral community model, co-occurrence network, Raup–Crick, soil protists

## Abstract

Understanding the maintenance of soil protists within forest ecosystems is crucial for comprehending ecosystem responses to climate change. A comprehensive analysis of soil samples from the Fengtongzhai National Reserve in China, utilizing high-throughput sequencing and network analysis, indicates that topsoil protistan communities predominantly comprise consumers, parasites, and plant pathogens. The principal phyla identified include Stramenopiles, Alveolates, Rhizaria (SAR), Cercozoa, Apicomplexa, and Ciliophora, with Monocystis, Rhogostoma, Cercomonas, and Globisporangium as the most prevalent genera. Although α diversity metrics did not reveal significant differences across various forest types, β diversity demonstrated notable distinctions, primarily influenced by soil pH, organic carbon content, and moisture levels. Complex co-occurrence networks were particularly evident in deciduous broadleaved and evergreen broadleaved mixed forests. The stability of these networks was higher in plantation forests compared with natural forests, with no significant differences observed among the three natural forest types studied. This finding challenges the reliability of using soil protists as indicators for forest soil health assessments. Stochastic processes, especially ecological drift, play a significant role in shaping these communities. In conclusion, the findings suggest that the mechanisms underlying the enhanced stability of co-occurrence networks of soil protists in plantations require further investigation. Additionally, the specific responses of soil protists to forest type highlight the necessity of incorporating multidimensional indicators in the evaluation of forest soil health and the effectiveness of ecological restoration efforts.

## 1. Introduction

Protists represent a diverse and essential group of microorganisms within soil ecosystems [1,2], playing a critical role in the soil food web [3]. They perform various functions, including acting as consumers, parasites, phototrophs, and mixotrophs [3,4,5,6], and they significantly influence nutrient cycling, microbial community assembly, and plant growth [5,7]. For instance, phototrophic soil protists contribute to carbon fixation and oxygen production [3,8], consumers regulate bacterial turnover and soil nutrient flux, and parasites can infect plants and animals [9].

Protist communities are sensitive to environmental changes, with their communities influenced by soil and climatic factors, as well as interactions with other organisms, such as bacteria, fungi, and invertebrates [7,10,11,12]. These influences exhibit variability across different protistan functional groups [7] and are further modulated by vegetation types [7,13], plant species identity [14], plant functional groups [15], phenological stages [16], plant health status [17], and soil compartments [18]. Differences in litter quality and root exudates among plant species lead to alterations in the soil conditions and microbial communities [19], thereby affecting protistan communities [16].

On a broader scale, the biogeography of protists in forest soils is influenced by factors such as soil pH, temperature, and precipitation [9]. Local environmental parameters also exert significant effects on protistan communities and ecosystem functions [20]. Soil pH influences nutrient availability and microbial interactions, while variations in temperature and precipitation affect protistan diversity by altering metabolic rates and soil moisture levels. Plant litter and root exudates contribute to the formation of microhabitats and the availability of soil organic matter (SOM), thereby shaping protistan communities [18]. The responses of individual trophic groups to identical factors vary, resulting in significant differences in abundance, species richness, and community composition across different land-use systems [21].

Soil organisms form complex ecological networks through interactions such as competition, mutualism, predation, and neutral associations [22,23]. Within these networks, protists consume bacteria and fungi, exerting top-down control, while themselves being preyed upon by higher trophic-level predators, including nematodes, collembolans, mites, earthworms, and insect larvae [24]. Soil invertebrates affect protist communities through predation, whereas bacteria and fungi influence them via bottom-up regulation [25]. Protists, acting as central hubs within the soil microbiome, are crucial to the connectivity, stability, and complexity of soil networks [26]. Although there is ongoing debate, molecular ecological networks can represent community complexity [16,27], with negative correlations often indicating abiotic variation (niche heterogeneity) within the environment [28,29]. A higher proportion of negative correlations suggests increased soil heterogeneity and a greater diversity of environmental niches [16]. A highly complex network is characterized by a greater number of nodes and edges and higher average degree and clustering, alongside reduced average path length and betweenness centrality [23,30]. This complexity enhances functional diversity and resilience [31].

Community assembly is shaped by both neutral and niche theories [32,33]. According to neutral theory, homogeneous dispersal (HD) results in uniform communities, whereas dispersal limitation (DL) leads to distinct community structures [34]. Niche theory posits that homogeneous selection (HS) drives community convergence, while variable selection (VS) promotes differentiation [32,35,36]. Additionally, drift (DR) introduces stochasticity through population fluctuations [37]. Geographical factors and plant species likely enhance community diversity by restricting dispersal and facilitating selection, although the specifics vary across different systems.

This study aimed to investigate the diversity and stability of soil protistan communities across various forest types. To achieve this objective, the following specific goals were established: (1) to analyze the α and β diversity of soil protistan communities in different forest types, (2) to identify the key environmental factors influencing the diversity and stability of these communities, (3) to compare the co-occurrence network stability of protistan communities across four distinct forest types, and (4) to quantify the role of community assembly processes, including both stochastic and deterministic processes, in shaping the structure of soil protistan communities. It is hypothesized that protistan diversity varies distinctly across forest types [20], and that their co-occurrence network patterns and community assembly processes are dependent on the forest type.

## 2. Materials and Methods

### 2.1. Sampling Sites

**Figure A1:**
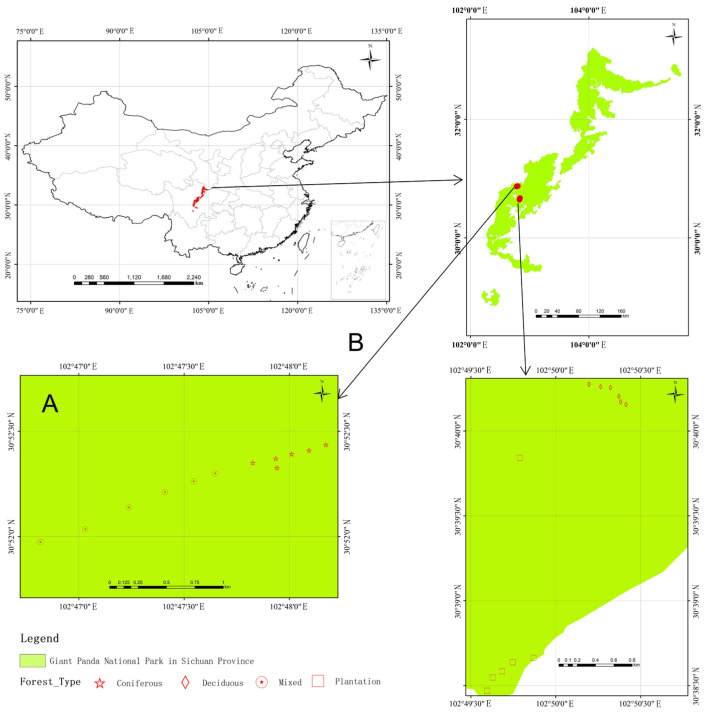
Schematic map showing the location of the study area and sampling points.

The study was conducted within the Fengtongzhai Nature Reserve, located in Baoxing County, Sichuan Province, China, with geographical coordinates extending from 102°48′ to 103°00′ E and from 30°19′ to 30°47′ N (Figure A1). This reserve encompasses approximately 390 square kilometers and is distinguished by its rugged ridges and narrow valleys, resulting in a diverse landscape with elevations ranging from 1000 to 4896 m. The region is subject to pronounced seasonal climatic variations, with spring occurring from April to June, summer and autumn from July to October, and winter from November to March. Meteorological data indicate that the average annual temperature varies between 5.9 and 7.2 °C, with humidity levels ranging from 79% to 83%. Precipitation is considerable, with an average annual rainfall of 730 to 1300 mm. July is the warmest month, with temperatures ranging from 15.1 to 16.3 °C, whereas January is the coldest, with temperatures between –4.0 and 2.7 °C.

The Fengtongzhai Nature Reserve exhibits vertical vegetation zonation [38,39]. At elevations below 1500 m above sea level, subtropical evergreen broadleaved forests predominate, with *Cinnamomum wilsonii* (Lauraceae) and *C. longepaniculatum* as the dominant tree species. As the elevation increases to between 1500 and 2000 m, the vegetation transitions to a mix of evergreen and deciduous broadleaved forests. This zone is characterized by the presence of prominent deciduous tree species such as *Pterocarya stenoptera* (Juglandaceae), *Betula* spp. (Betulaceae), and *Acer* spp. (Seapindaceae). Between the altitudes of 2000 and 2900 m, the forest composition shifts to a mix of coniferous and deciduous trees, with notable species including *Tsuga chinensis* (Pinaceae) and *Pinus armandii* (Pinaceae), alongside the deciduous *Betula* spp. Above 2900 m, conifer forests take over, and beyond 3500 m, the landscape transitions to shrubs and grasslands, showcasing the varied plant life that adapts to the changing elevations within the reserve.

### 2.2. Soil Sampling

Within this study, soil sampling was conducted in October 2021 across four selected forest types: coniferous (Con) forest, deciduous broadleaved (Dec) forest, mixed forest consisting of both deciduous broadleaved and evergreen broadleaved species (Mix), and coniferous forest plantation (Pla). In each forest type, 18 sampling plots were established, considering the dominant tree species, canopy cover, and primary understory species. Each plot comprised three quadrants, each measuring 5 m × 5 m, from which soil samples (0–20 cm deep) were collected using a soil auger with a 5 cm diameter. Samples were obtained from five locations along an S-shaped trajectory following the removal of surface debris (including leaves, dry vegetation, and litter) to ensure uniform mixing and accurate representation of the area. The collected soil samples were placed into sterilized press seal bags and stored in an icebox before being promptly transported to the laboratory for further analysis. The soil was then divided into two portions for distinct analytical purposes: one portion was naturally air-dried to assess soil physicochemical characteristics, while the other portion was preserved for additional analyses.

### 2.3. Soil Physicochemical Property Determination

The physicochemical properties of the soil were evaluated using a standardized procedure outlined in previous research [40]. In particular, soil water content (SWC) was assessed with 10 g of field-moist samples subjected to 105 °C for 24 h. A glass electrode pH meter was employed to measure soil pH, maintaining a soil-to-water ratio of 1:2.5. The high-temperature external heat dichromate oxidation capacity method was utilized to determine the soil organic carbon (SOC) content. For the contents of total nitrogen (TN) and available nitrogen (AN) in the soil, the Kjeldahl digestion and alkaline hydrolysis methods were applied, respectively. To measure total phosphorus (TP) content in the soil, the Mo–Sb anti-spectrophotometric technique was used. Available phosphorus (AP) was extracted with an HCl-NH_4_F solution and analyzed via the molybdenum–antimony resistance colorimetric method. The ratios of SOC to soil TN (C/N), SOC to soil TP (C/P), and soil TN to TP (N/P) were calculated.

### 2.4. DNA Extraction and Library Preparation

Soil protistan DNA was extracted from 0.5 g soil using the OMEGA Soil DNA Kit (M5635-02) (Omega Bio-Tek, Norcross, GA, USA), following the manufacturer’s instructions, and stored at −20 °C prior to further analysis. DNA extraction was performed in triplicate for each soil sample. The quantity and quality of the extracted DNA were measured using a NanoDrop NC2000 spectrophotometer (Thermo Fisher Scientific, Waltham, MA, USA) and agarose gel electrophoresis, respectively. The V9 region of the 18S rRNA gene was amplified from the extracted DNA using the universal eukaryotic primers (1389F/1510R) [1,13,16]. PCR was performed as follows: initial heating to 94 °C for 4 min, followed by 25 thermal cycles (30 s at 94 °C, 60 s at 57 °C, and 90 s extension at 72 °C) and concluded with a 10 min final auto-extension at 72 °C [13]. PCR products were purified using the Axygen DNA Gel Extraction Kit (Axygen Biosciences, Union City, CA, USA), and amplicons were pooled in equal amounts. Paired-end sequencing (2 × 300 bp) was performed using standard protocols by Shanghai Personalbio Technology Co., Ltd. (Shanghai, China) on the Illumina MiSeq sequencing platform (Thermo Scientific, Wilmington, DE, USA, 2 × 300 base pairs (bp)).

### 2.5. Bioinformatics Processing

Sequencing data processing and taxonomic annotation were performed according to the protocol as described elsewhere [41]. Sequences were quality-filtered, denoised, and merged, and chimeric sequences were removed via a DADA2 plugin in QIIME 2. Amplicon sequence variants (ASVs) with 100% similarity were identified using UPARSE [42] and classified against the PR^2^ database [36] (v4.14.0). To obtain exclusive protist data, non-protist sequences (fungi, Metazoa, unidentified Opisthokonta, Streptophyta, Rhodophyta, and unclassified eukaryotes) were removed from all samples using QIIME 2 (taxa filter-table/seq).

### 2.6. Statistical Analysis

All statistical analyses were performed using the R (version 4.3.2) unless otherwise specified. The nonparametric Kruskal–Wallis test with false discovery rate correction (FDR-adjusted *p* < 0.05) was used to test the differences in the α diversity of protists and their functional taxa in different forest types using R (v. 4.1.3). Then, Spearman correlation coefficients between soil protists (including the relative abundance of dominant taxa and α diversity) and soil physicochemical properties were examined. The distinct structure of the protistan communities across forest types was evaluated by beta diversity and visualized via principal coordinate analysis (PCoA) using ASV data. The statistical significance among samples collected from five different forest types were examined by performing permutational multivariate analysis of variance (PERMANOVA) with 999 random permutations (*p* < 0.05) using the adonis 2 function of the package “vegan” of R. Specific association taxa for a given forest type were further examined via indicator species analysis [43] using in R (version 4.3.2). Statistically robust Spearman correlations (|ρ| > 0.6 and *p* < 0.01) were kept to construct the networks [23]. Co-occurrence networks and topological features were analyzed using the “meconetcomp” and “igraph” packages [44]. The commonly used topological features of subnetworks, such as node number, edge number, average degree, clustering coefficient, and density [31,45,46] were subsequently calculated. Additionally, the vulnerability [47] and stability ($network\;stability=\frac{Negative\;cohesion}{Positive\;cohesion}$) of co-occurrence networks were quantified.

The ecological processes were quantified using a method named Infer Community Assembly Mechanisms via the phylogenetic-bin-based null model (iCAMP) (version 1.3.4) [48,49]. Firstly, iCAMP assigned the observed taxa into phylogenetic closed groups (bins). Then both the within-bin beta Net Relatedness Index (βNRI) and the modified Raup–Crick metric (RC) were calculated to estimate the relative contribution of homogeneous selection (HS; βNRI < −1.96), variable selection (VS; βNRI > 1.96), homogeneous dispersal (HD; RC < −0.95 and |βNRI| ≤ 1.96), dispersal limitation (DL; RC > 0.95 and |βNRI| ≤ 1.96), and drift (DR; |RC| ≤ 0.95 and |βNRI| ≤ 1.96) in governing community assembly [32]. Finally, a structural equation model (SEM) was used to explore the causal relationships among forest types, soil physicochemical properties, and protistan community compositions.

## 3. Results

### 3.1. Soil Physicochemical Properties

Significant differences in soil water content (Kruskal–Wallis rank sum test: *χ^2^* = 29.048, *df* = 3, *p* < 0.001), electrical conductivity (Kruskal–Wallis rank sum test: *χ^2^* = 28.699, *df* = 3, *p* < 0.0001), pH (Kruskal–Wallis rank sum test: *χ^2^* = 39.237, *df* = 3, *p* < 0.0001), SOC content (Kruskal–Wallis rank sum test: *χ^2^* = 20.441, *df* = 3, *p* = 0.0001), and total phosphorus content (Kruskal–Wallis rank sum test: *χ^2^* = 16.426, *df* = 3, *p* = 0.0009) were observed across forest types. Additionally, soil stoichiometry including C/N (Kruskal–Wallis rank sum test: *χ^2^* = 28.676, *df* = 3, *p* < 0.0001), C/P (Kruskal–Wallis rank sum test: *χ^2^* = 207.72, *df* = 3, *p* < 0.0001) and N/P (Kruskal–Wallis rank sum test: *χ^2^* = 15.928, *df* = 3, *p* = 0.0012) varied greatly across forest types.

### 3.2. Taxonomic and Functional Group Composition

**Figure 1:**
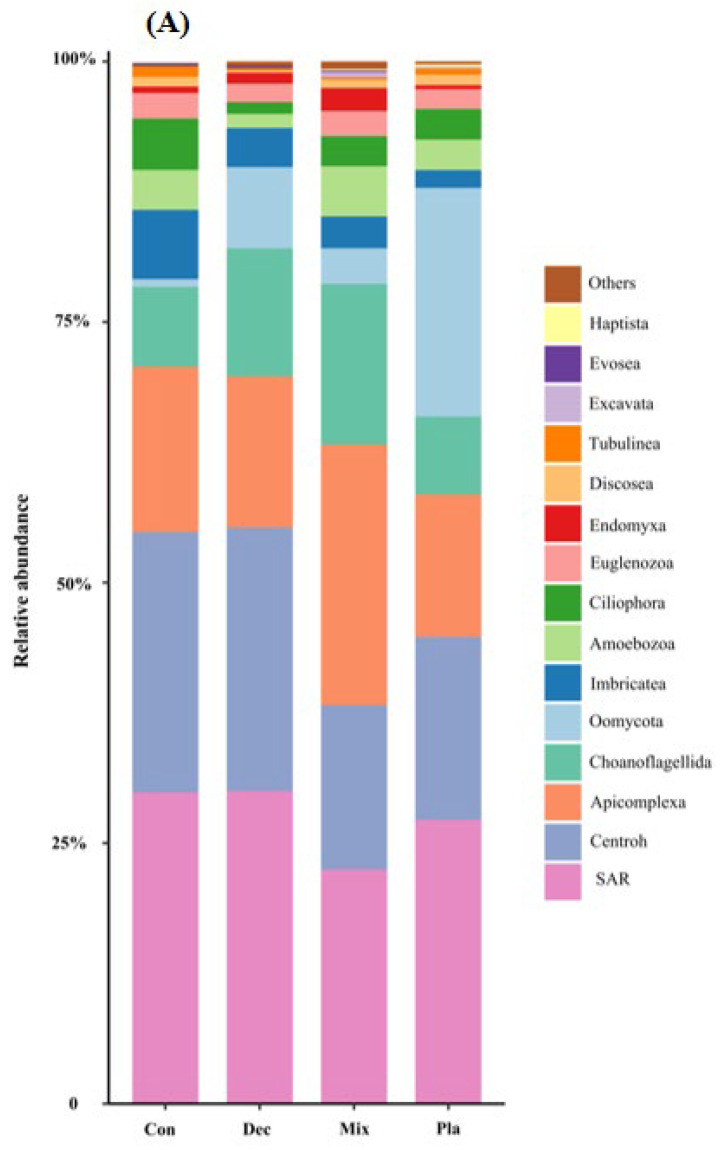

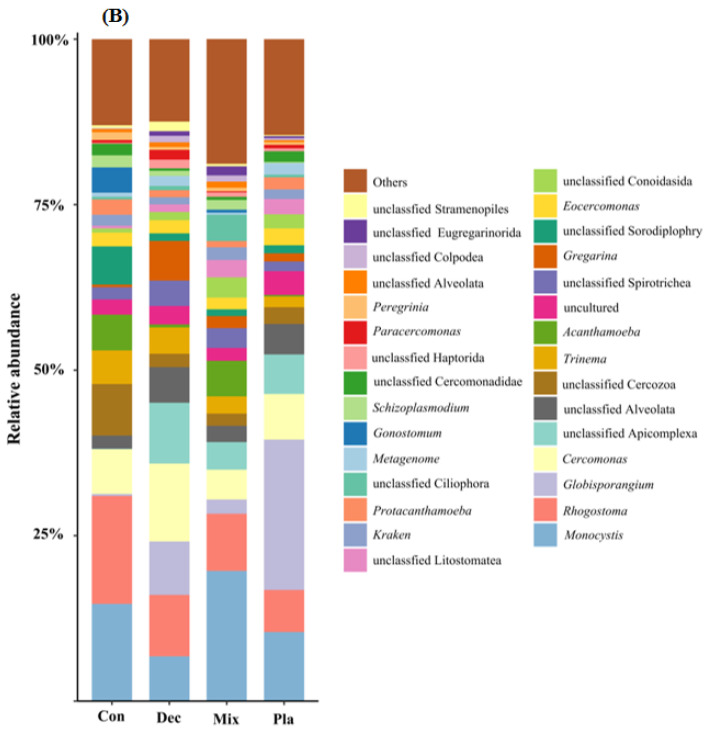
Relative amplicon sequence variant (ASV) abundance of soil protists at the phylum (**A**) and genus (**B**) level in in coniferous forest (Con), deciduous broadleaved forest (Dec), natural deciduous broadleaved and evergreen broadleaved mixed forest (Mix), and plantation forest (Pla). Any taxa that have less than 1% cumulative mean relative abundance were grouped under the category “Other” in the figures.

**Figure A2:**
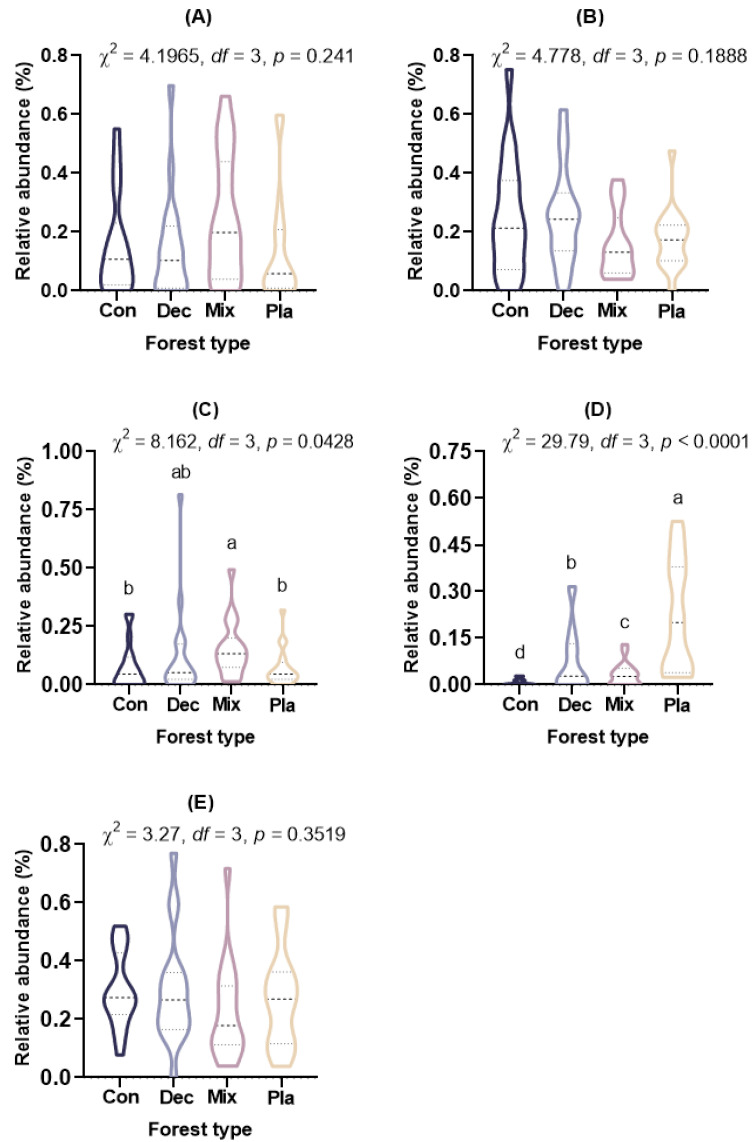
The relative abundance (%) of dominant soil protistan phyla in topsoil of typical forest types in Fengtongzhai National Reserve, China. The horizontal lines within the violin plots represent the median, upper, and lower quartiles. (**A**) Apicomplexa; (**B**) Cercozoa; (**C**) Ciliophora; (**D**) Oomycota; (**E**) Stramenopiles, Alveolates, and Rhizaria (SAR) supergroup. Different lowercase letters indicated significant differences between different forest types (*p* < 0.05).

**Figure A3:**
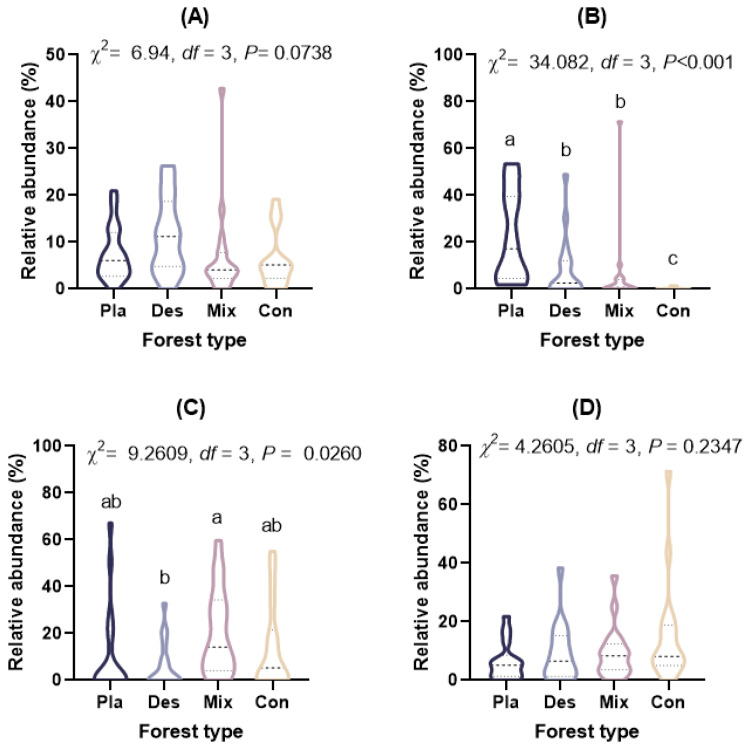
The relative abundance (%) of (**A**) *Cercomonas*, (**B**) *Globisporangium*, (**C**) *Monocystis*, and (**D**) *Rhogostoma* in the topsoil of typical forest types in Fengtongzhai Reserve, China. The horizontal lines within the violin plots represent the median, upper, and lower quartiles. Different lowercase letters indicated significant differences between different forest types (*p* < 0.05).

At the phylum level (Figure 1A), the topsoil protist community was dominated by the SAR supergroup (22.43–29.98%), followed by Cercozoa (15.82–25.32%), Apicomplexa (13.59–24.88%), and Ciliophora (7.48–15.46%). The relative abundance of Ciliophora in mixed forests was significantly higher than in other forest types, except for deciduous broadleaved forests. There was a significant variation in the relative abundance of Oomycota across different forest types (Figure A2). The relative abundance of Oomycota was positively correlated with soil pH, while it was negatively correlated with latitude and elevation. At the genus level (Figure 1B), soil protists in topsoil were dominated by *Monocystis* (6.40–20.49%), followed by *Rhogostoma* (6.51–15.13%), *Cercomonas* (6.20–11.55%), and *Globisporangium* (0.3–22.47%). There were significant differences in the relative abundance of *Globisporangium* and *Monocystis* across the distinct forest types (Figure A3).

**Figure 2:**
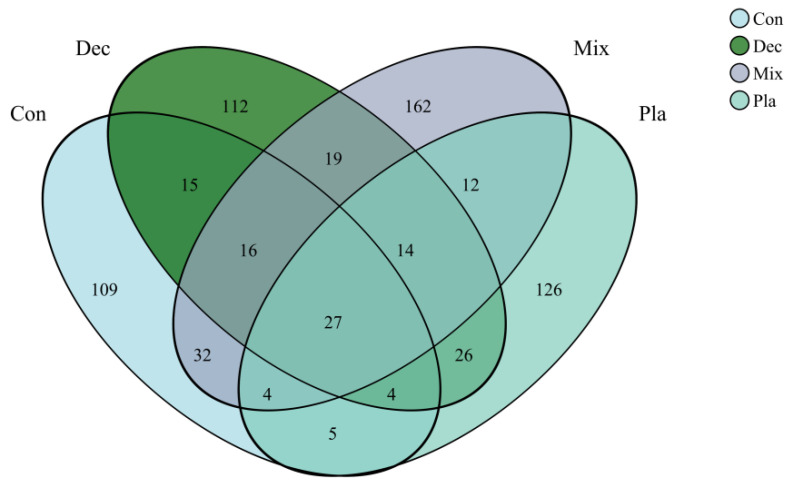
Venn diagram of soil protists at amplicon sequence variant (ASV) level, illustrating the shared and specific soil protists in in coniferous forest (Con), deciduous broadleaved forest (Dec), natural deciduous broadleaved and evergreen broadleaved mixed forest (Mix), and plantation forest (Pla).

As illustrated in the Venn diagram (Figure 2), numerous amplicon sequence variants (ASVs) were unique to specific forest types, while the majority of the protist communities comprised shared ASVs, which represented a minor proportion of the total ASV count. *Monocystis agilis* exhibited a strong and significant association with plantation forests. Similarly, *Rhogostoma miunus* was associated with mixed forests, and *Cercomonas celer* and *Rhogostoma miunus* with coniferous forests.

### 3.3. Diversity

**Figure 3:**
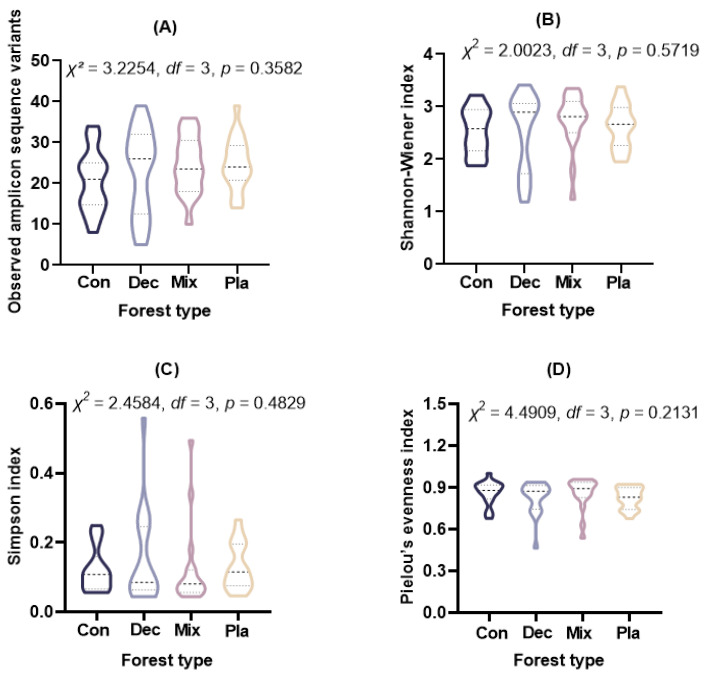
The (**A**) observed amplicon sequence variants, (**B**) Shannon–Wiener index, (**C**) Simpson index, and (**D**) Pielou’s evenness index for soil protist communities in coniferous forest (Con), deciduous broadleaved forest (Dec), natural deciduous broadleaved and evergreen broadleaved mixed forest (Mix), and plantation forest (Pla). The results above the small boxes in each boxplot are based on the nonparametric Kruskal–Wallis test. The horizontal lines within the violin plots represent the median, upper, and lower quartiles.

Alpha diversity metrics, including observed ASVs (Kruskal–Wallis rank sum test: χ^2^= 3.2254, *df* = 3, *p* = 0.3582), Shannon–Wiener index (Kruskal–Wallis rank sum test: χ^2^= 2.0023, *df* = 3, *p* = 0.5719), Simpson index (Kruskal–Wallis rank sum test: χ^2^ = 2.4584, *df* = 3, *p* = 0.4829), and Pielou’s evenness index (Kruskal–Wallis rank sum test: χ^2^ = 4.4909, *df* = 3, *p* = 0.2131), indicated no significant differences among the forest types examined (Figure 3).

**Figure 4:**
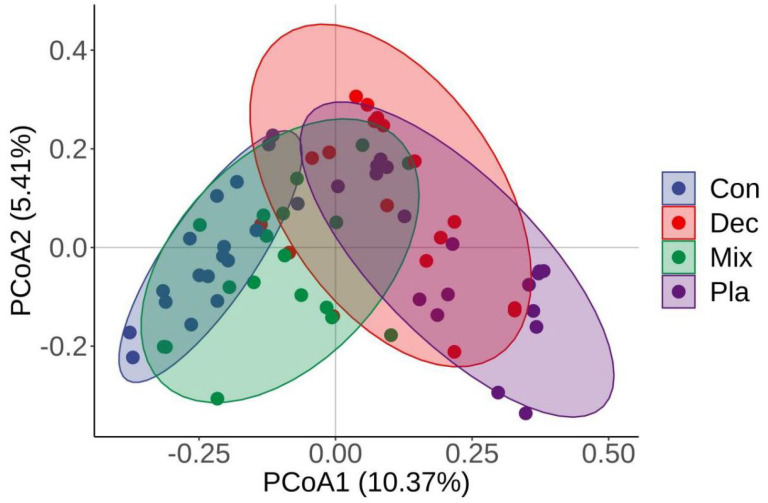
Principal coordinate analysis (PCoA) plots comparing soil protistan communities in coniferous forest (Con), deciduous broadleaved forest (Dec), natural deciduous broadleaved and evergreen broadleaved mixed forest (Mix), and plantation forest (Pla) based on the Bray–Curtis distance.

The principal coordinate analysis (PCoA) further corroborated that the soil protistan community in the topsoil layer can be categorized into four distinct groups (Figure 4). The first two PCoA axes accounted for 10.37% and 5.41% of the variations in the soil protistan community in the topsoil layer. ANOSIM, PERMANOVA, and MRPP (Table 1) further verified that the protistan community structures in bulk soils in the upper layer differed significantly among forest types. The “Envfit” analysis supported that the critical driving factors of the soil protistan community in the topsoil layer were pH, SWC, EC, TP, SOC, C/N, C/P, and N/P (Table 2).

### 3.4. Co-Occurrence Networks

**Figure 5:**
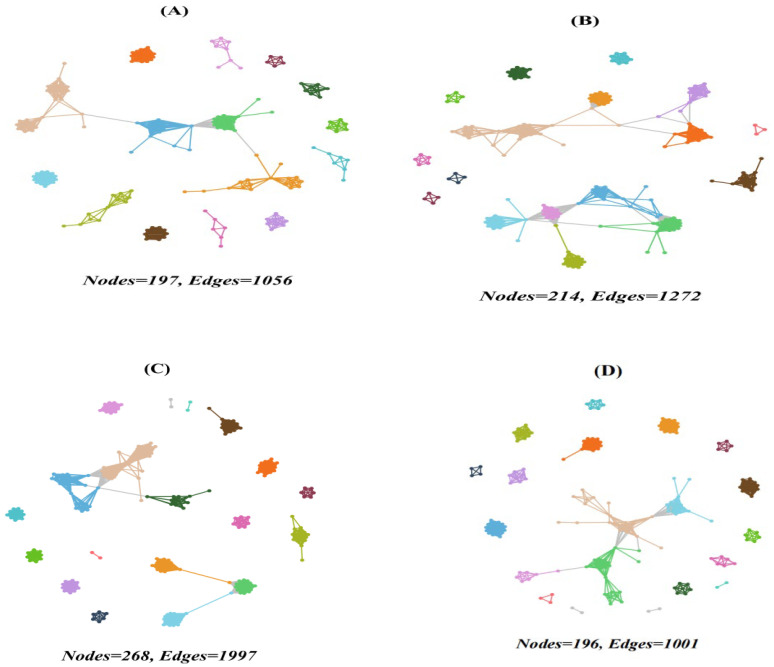
Network co-occurrence of abundant soil protists in the topsoils of coniferous forest (**A**), deciduous broadleaved forest (**B**), natural deciduous broadleaved and evergreen broadleaved mixed forest (**C**), and plantation forest (**D**). Each connection stands for the SparCC correlation with the magnitude of r > 0.6 (positive correlation) or r < −0.6 (negative correlation) and was statistically significant (*p* < 0.001). Each node represents different soil protistan ASVs, and the size of the node is proportional to the number of connections (degree).

**Figure 6:**
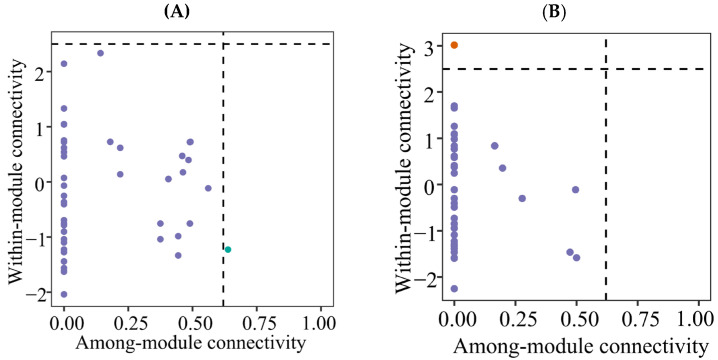

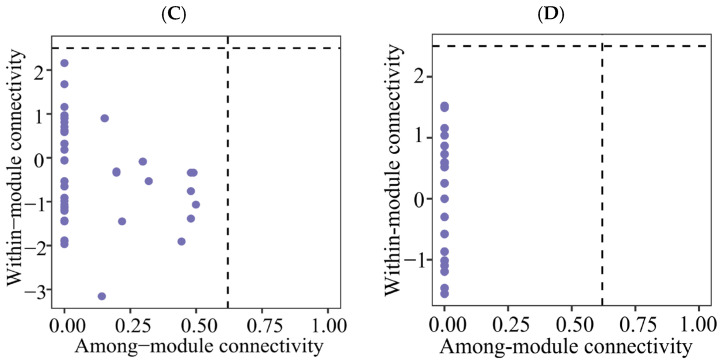
Zi-Pi plot showing the distributions of ASVs based on their topological roles of soil protistan communities in the topsoils of coniferous forest (**A**), deciduous broadleaved forest (**B**) natural deciduous broadleaved and evergreen broadleaved mixed forest (**C**), and plantation forest (**D**).

The co-occurrence network analysis revealed complex interactions among protistan taxa in the topsoils of forests (Figure 5 and Figure 6). The deciduous broadleaved and evergreen broadleaved mixed forest had the most intricate networks, characterized by a higher degree of connectivity (Figure 5C) and average degree. Additionally, the network vulnerability was, on average, lower in deciduous broadleaved and evergreen broadleaved mixed forest in comparison with other forest types.

### 3.5. Community Assembly Processes

**Figure 7:**
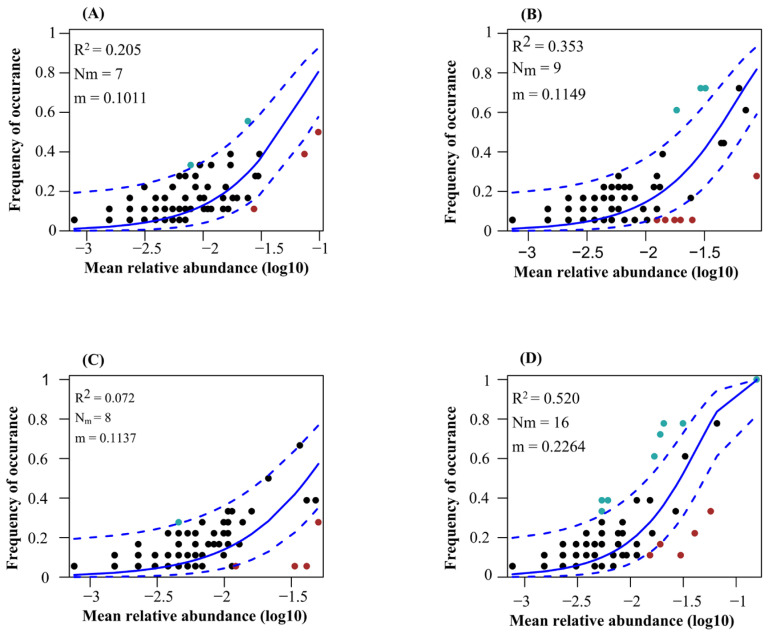
Fit of Sloan’s neutral community model for soil protistan ASVs in the topsoil of coniferous forest (**A**), deciduous broadleaved forest (**B**), natural deciduous broadleaved and evergreen broadleaved mixed forest (**C**), and plantation forest (**D**). Solid blue lines show the best fit; dashed blue lines show the 95% confidence intervals. ASVs occurring more or less frequently than predicted are shown in red and green, respectively. R^2^ indicates the goodness of fit; m value indicates the migration rate.

**Figure 8:**
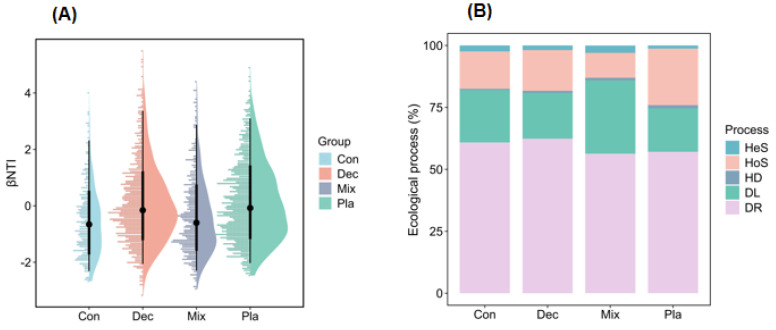
Assembly processes of soil protistan communities in the upper layer of coniferous forest (Con), deciduous broadleaved forest (Dec), natural deciduous broadleaved and evergreen broadleaved mixed forest (Mix), and plantation forest (Pla). (**A**) The βNTI values and (**B**) the relative contributions of different assembly processes, including heterogeneous selection (Hes), heterogeneous selection (HoS), homogenizing dispersal (HD), dispersal limitation (DL), and ecological drift (DR).

The neutral community model explained the higher variation of protistan communities in the topsoil of plantation forest in comparison with natural forests (Figure 7). Additionally, the neutral community model explained the higher variation of protistan communities in the topsoil of deciduous broadleaved forest, followed by coniferous forest (Figure 7). The betaNTI values of soil protistan communities were between −2 and 2, indicating the dominant role of stochastic processes (Figure 8A). The quantitative estimates of the relative contribution of assembly processes showed that stochastic processes, particularly ecological drift and dispersal limitation, were the dominant drivers of soil protistan communities in the topsoil of coniferous forest, deciduous broadleaved forest, deciduous broadleaved and evergreen broadleaved mixed forest, and plantation forest (Figure 8B).

## 4. Discussion

In the present study, the patterns of diversity, co-occurrence networks, and assembly processes of soil protistan communities in the upper soil layer across four distinct forest types, including coniferous forest, deciduous broadleaved forest, mixed coniferous and broadleaved forest, and plantation forest, were examined. Our findings provide crucial insights into the influence of environmental factors on the structure and dynamics of protistan communities in these ecosystems.

### 4.1. Taxonomical and Functional Composition

Our results corroborate previous studies indicating that soil protistan communities are predominantly composed of consumers [21,50], particularly those from the SAR supergroup [2]. This dominance is associated with their role in regulating other soil biotas [5,50]. The observed high relative abundance of the SAR supergroup can be attributed to their diverse metabolic strategies, which enable them to occupy various ecological niches. For instance, stramenopiles include both heterotrophic and phototrophic organisms, allowing them to exploit different energy sources [51]. SAR protists demonstrate adaptability to changing conditions in the topsoil, with Alveolates, such as ciliates, thriving under variable moisture and nutrient conditions [52], and Rhizaria, particularly Cercozoans, acting as predators of bacteria and small organisms, thus maintain microbial balance. This predatory role of SAR taxa is crucial for soil health and ecosystem function. Many engage in symbiotic relationships, such as stramenopiles with plant roots, boosting nutrient uptake and plant health [53]. The SAR supergroup’s extensive evolutionary history and diversification have led to high species richness, particularly in forest soils [54], contributing to their dominance.

Our findings partly support the idea that soil protistan communities vary with forest type [20,21,55]. Notably, *oomycotes* are more prevalent in plantation forests, likely due to factors like the higher incidence of phytophthora (e.g., *Eucalyptus* or *Pinus*) compared with mixed or natural forests [56,57], attributed to more suitable hosts. Natural forests typically have a great variety of plant species, which can limit the presence of specific host plants of *oomycotes*. In contrast, plantation forests often have unique microclimatic conditions such as extensive litter cover that keeps soil moisture high, promoting the growth of *oomycotes*, which thrive in moist conditions Coniferous forests, with their lower nutrient levels, are less favorable for saprophytic *oomycotes* compared with mixed and broadleaved forests, reducing the risk of *oomycotes’* proliferation.

### 4.2. Diversity Patterns

Changes in forest type did not significantly affect α diversity but altered soil protistan communities. Previous studies have shown mixed results regarding the α diversity of soil protists between natural and plantation forests, indicating species-specific effects, with others noting significant differences [20,21,55]. This discrepancy may be attributed to differences in the soil types and plant communities examined. The observed significant variations in the β diversity of soil protistan communities across forest types are mainly due to environmental factors like soil pH, nutrient availability, and moisture content, which influence protistans’ composition and diversity [21]. Specifically, soil pH and nutrient gradients are known to influence the composition and diversity of protistan communities by affecting their metabolic and physiological processes [58]. Additionally, distinct vegetation types contribute to the heterogeneity in the forest floor, which, in turn, affects the distribution and abundance of soil protists [5]. Furthermore, ecological processes such as competition, predation, and symbiotic relationships also help to explain the observed β diversity. For instance, protists serve as both predators and prey within the soil food web, influencing population dynamics through trophic interactions [59]. The presence of specific plant species and their associated root exudates can further modify the microbial habitat, thereby impacting the structure of protistan communities [60]. Another critical factor to consider is the role of spatial and temporal heterogeneity in shaping protistan community diversity. Forest ecosystems are characterized by a mosaic of micro-habitats, each with unique physicochemical conditions that support diverse soil protistan communities [61]. Seasonal changes also influence the soil protistan community [62,63]. Moreover, anthropogenic factors such as forest management practices and land-use changes can significantly impact diversity of soil protistan communities. For example, coniferous plantation forests often involve monoculture practices and land-use changes that reduce habitat complexity and soil organic matter, which can negatively affect the diversity of soil protistan communities compared with more diverse natural forests [64]. In addition to these environmental and ecological factors, evolutionary processes such as dispersal and speciation also contribute to the observed patterns of β diversity. Dispersal limitation can lead to distinct protistan communities in geographically isolated forest types, while local adaptation and speciation can result in unique assemblages adapted to specific environmental conditions [65]. To further elucidate the mechanisms driving β diversity in soil protistan communities, future research should focus on high-resolution temporal and spatial sampling, combined with advanced molecular techniques such as metagenomics and transcriptomics. These approaches can provide deeper insights into the functional roles of protists and their interactions with other soil organisms [2]. Additionally, experimental studies manipulating key environmental variables such as soil pH, moisture, and nutrient levels can help establish the causal relationships between these factors and protistan diversity. Long-term monitoring of protistan communities in response to forest management practices can provide valuable information for sustainable forest management and conservation strategies [66]. In conclusion, the β diversity of soil protistan communities across different forest types is influenced by a complex interplay of environmental, ecological, and evolutionary factors. Understanding these relationships is crucial for predicting the responses of soil microbial communities to environmental changes, and for maintaining ecosystem health and resilience.

Soil properties were found to be the most important factor shaping soil protistan assemblages in bulk soils. Similar results were reported by other studies [3,5,9,16,22,67]. Variations in the soil protistan community can be attributed to differences in pH, moisture, and soil type across sampling sites [16], as well as soil pore size, moisture, pH, and nutrient content [3,5,9,22]. Key drivers of soil protistan community composition and diversity include soil physicochemical properties such as pH, organic carbon content, nutrient levels, and moisture [2,3,68]. At the local scale, soil pH and electrical conductivity have been reported as determinants of the density, diversity, species composition, distribution, and activity of protists [3,69]. For instance, higher pH values in deciduous broadleaved forests were correlated with greater protistan diversity, supporting the notion that neutral to slightly alkaline conditions favor a wider range of protists. SOM, a critical factor for nutrient availability, is positively associated with protistan richness across all forest types. Water availability controls the development of plant pathogenic o*omycetes*. Soil moisture also played a significant role, particularly in coniferous and mixed forests, where water availability can be more variable. Soil pH significantly and negatively affects the diversity and composition of phototrophs [7]. Total nitrogen is another edaphic factor with significant effects on the diversity and structure of the phototrophic protists [7].

Diverse protist communities significantly enhance soil nutrient cycling [60] and carbon sequestration [21]. They regulate bacterial and fungal populations through predation, improving nutrient mineralization and organic matter decomposition [70]. This process reduces competition among microbial decomposers and makes nutrients more accessible to plants [60]. Protists also improve the soil structure by excreting extracellular polymeric substances that bind soil particles, enhancing soil aeration, water retention, and root penetration [71], which benefits plant growth and soil health [72]. Additionally, their symbiotic associations with soil organisms like mycorrhizal fungi boost nutrient uptake and stress resilience in plants [73].

Protists aid mycorrhizal fungi in colonizing plant roots, enhancing the plants’ nutrient and water absorption, especially phosphorus [74], which is vital in nutrient-poor soils. In the face of climate change, protists play a key role in carbon sequestration by forming stabilizing SOM through their predation on bacteria and fungi, leading to the release of microbial residues that are key precursors to stable SOM [75]. This process helps capture carbon in the soil, reducing atmospheric CO_2_ concentrations [76]. Protists’ adaptability to various environmental conditions also makes them useful indicators of soil health and ecosystem stability [62], as they respond sensitively to changes in soil moisture, temperature, and pH, providing early warnings of disturbances [77]. Future research should further explore the molecular mechanisms underlying protist–microbe–plant interactions. Advanced metagenomic and meta-transcriptomic techniques can uncover the functional genes and pathways involved in these interactions, providing insights into how protists influence soil biochemical cycles at the molecular level [78].

### 4.3. Co-Occurrence Networks

Forest type was found to be a good predictor of the soil protistan community’s occurrence network pattern. The co-occurrence network analysis revealed complex interactions among protistan taxa in each forest type. Specifically, mixed forests had the most intricate networks, characterized by a high degree of connectivity and numerous positive and negative associations. The complexity of soil protistans’ co-occurrence network likely reflects the diverse niches and resources available in these forests. In coniferous forests, the co-occurrence networks were simpler and more modular, suggesting that protistan interactions are more compartmentalized and possibly driven by specific environmental constraints. The soil protistan community in plantation forests exhibited the least complex networks, possibly due to reduced habitat heterogeneity and simplified ecological interactions. Module hubs and connectors may represent keystone species in an ecosystem [79,80]. In the present study, rare taxa can be putative keystone species.

One interesting finding of the present study is that the co-occurrence network stability of soil protistan communities in plantation forests was higher than in natural forests, whereas no significant differences were observed among the three examined natural forest types. There are several likely reasons why the stability of soil protistan communities is higher in plantations compared with natural forests. Firstly, plantations often have a more homogenous environment due to the use of monocultures or a limited number of tree species, resulting in more consistent environmental conditions such as soil pH, moisture, and nutrient availability, which can create a more stable and predictable environment for soil protistan communities, enhancing network stability. Secondly, plantations are typically managed with regular tending including pruning, weeding, and pest control, which can reduce environmental variability and disturbance, promoting stable microbial communities. Additionally, natural forests experience a range of disturbances such as tree falls, herbivory, and natural succession, which can disrupt microbial networks and lead to lower stability, while managed plantations provide a more consistent habitat for soil protists. Furthermore, plantations may receive regular nutrient inputs, reducing competition for resources among protists, and less soil heterogeneity, promoting stable protistan networks. Microbial interactions in plantations may also be more predictable and stable due to simpler food webs and lower pathogen pressure. Lastly, plantations often have a more consistent microclimate, resulting in less variation in the microclimate conditions compared with natural forests with diverse vegetation structures. These factors contribute to the higher stability of soil protistan communities in plantations compared with natural forests.

### 4.4. Community Assembly Processes

Our study demonstrates that stochastic processes significantly influence the structuring of protistan communities in forest soils, with the extent of this impact varying across different forest types. This finding corroborates previous research suggesting that stochastic factors play a role in shaping soil protistan communities [81]. The results underscore the complex nature of protistan community assembly, highlighting the interplay between deterministic and stochastic processes, which are modulated by the forests’ environmental conditions and management practices. Additionally, elevation is found to have a substantial effect on these processes [82]. In deciduous broadleaved forests, environmental filtering is predominant due to stable micro-habitats and diverse plant life, which create predictable niches that favor specific protistan taxa. This is further supported by stable seasonal patterns and abundant organic matter [83,84]. Future research should investigate the specific micro-habitat characteristics that drive strong environmental filtering and the effects of seasonal changes on these communities. Conversely, in coniferous forests, characterized by harsh conditions such as acidic soils, stochastic processes are more influential. This suggests that random events, including dispersal limitation and ecological drift, become more prominent when niche formation is less favorable [85,86]. Investigating the impact of abiotic stressors on protistan diversity, alongside the roles of soil chemistry and microclimate in these forests, is of significant academic interest. Mixed forests, which balance deterministic and stochastic processes, provide an opportunity to examine the interplay between these mechanisms. The heterogeneity of these forests offers a variety of niches and random disturbances, contributing to a dynamic equilibrium [87,88]. Gaining insights into these interactions within mixed forests can enhance forest ecosystem management strategies. In contrast, plantation forests, heavily influenced by human activities, demonstrate how management practices such as monoculture planting and soil amendments can disrupt the natural assembly of microbial communities, thereby favoring stochastic processes [89,90]. This underscores the importance of sustainable forestry practices that prioritize the conservation of microbial diversity.

In the soils of switchgrass (*Panicum virgatum*), protistan communities are predominantly shaped by homogenous selection and dispersal limitation, whereas in forest soils, community assembly varies according to functional group. For instance, the distribution of Cercozoa is more affected by homogenizing dispersal, while Apicomplexa is influenced by ecological drift [9]. These findings underscore the intricate dynamics of microbial communities and the imperative to account for both biotic and abiotic factors in ecosystem management. Notably, the differential effects observed among functional groups underscore the importance of considering ecological roles in the study of protist community assembly. To further elucidate these mechanisms, it is crucial to integrate multi-omics approaches, such as metagenomics, transcriptomics, and metabolomics, with environmental data. This integrative methodology can yield a more comprehensive understanding of how specific environmental variables and microbial interactions influence community assembly processes. For example, metagenomics can uncover the genetic potential of protist communities, while transcriptomics can illuminate their active functions under varying environmental conditions. Metabolomics, in contrast, can provide insights into the metabolic exchanges between protists and other soil organisms, thereby highlighting potential mutualistic or antagonistic interactions. Furthermore, long-term ecological studies are required to capture the temporal dynamics and successional changes within protist communities. Moreover, the integration of spatially explicit models can significantly enhance our comprehension of dispersal limitation and its impact on community assembly processes. Such spatial models are capable of simulating the movement of protists across heterogeneous landscapes, taking into account variables such as soil texture, connectivity, and dispersal barriers. These models are instrumental in predicting the potential effects of land-use changes or habitat fragmentation on the dispersal and distribution patterns of protist communities [78]. Additionally, it is imperative to consider the broader ecological and evolutionary contexts within which these communities operate. Protists do not exist in isolation; they engage in interactions with a diverse array of soil organisms, including bacteria, fungi, and plant roots. A comprehensive understanding of these interactions and their implications for community assembly necessitates an integrative approach that encompasses the entire soil microbiome and its interactions with the abiotic environment. For instance, examining the co-occurrence patterns of protists alongside other microorganisms can uncover potential symbiotic relationships or competitive dynamics that shape community composition and assembly.

This study highlights that the assembly of protistan communities is a complex process shaped by an intricate interplay of deterministic and stochastic factors. Future research should focus on identifying the specific drivers of these processes across various forest types and examining their implications for ecosystem functioning and resilience. Employing metagenomic and transcriptomic methodologies could yield deeper insights into the functional roles of protistan communities and their responses to environmental changes. Moreover, it is essential to consider the broader ecological and evolutionary ramifications of our findings. Understanding the equilibrium between deterministic and stochastic processes in microbial community assembly can inform broader ecological theories and enhance biodiversity conservation strategies. By advancing our comprehension of the mechanisms underlying microbial diversity, we can improve our ability to predict and mitigate the impacts of environmental changes on forest ecosystems.

## 5. Conclusions

In conclusion, this study underscores the ecological and evolutionary importance of microbial community assembly, highlighting the interplay between deterministic and stochastic processes. Our findings reveal similar α diversity but distinct β diversity across different forest types, with a particularly intricate co-occurrence network observed in mixed deciduous and evergreen broadleaved forests. Drift plays a significant role in shaping protistan communities within forest topsoil. This research advances our comprehension of the patterns and environmental determinants of soil protistans’ diversity, co-occurrence networks, and assembly processes, thereby contributing to forest biodiversity conservation efforts. Future research should focus on elucidating the functional roles of soil protists within forest ecosystems and assessing the impacts of climate change and anthropogenic disturbances on these vital communities. Additionally, the specific responses of soil protists to different forest types highlight the necessity of incorporating multidimensional indicators in the evaluation of forest soil health and the effectiveness of ecological restoration efforts.

## Figures and Tables

**Table 1:** The overall similarities in the soil protistan community across diverse forests were examined using three nonparametric statistical methods.

Object	Adonis			ANOSIM		MRPP			
	*F*	*R* ^2^	*Adjusted*-*p*	*R*	*Adjusted*-*p*	*A*	Observed-δ	Expected*-δ*	*Adjusted*-*p*
Among group	3.3321	0.1282	0.001	0.3586	0.001	0.0488	0.8766	0.9216	0.001
Con vs. Dec	3.8148	01009	0.001	0.4608	0.001	0.0393	0.9245	0.8881	0.001
Con vs. Mix	1.9036	0.0530	0.001	0.1855	0.001	0.0131	0.9286	0.9164	0.002
Con vs. Pla	5.7874	0.1455	0.001	0.7138	0.001	0.0647	0.9121	0.8531	0.001
Dec vs. Mix	2.1576	0.0597	0.001	0.1876	0.001	0.0167	0.9153	0.9001	0.001
Dec vs. Pla	2.7590	0.0751	0.001	0.2155	0.001	0.0264	0.8595	0.8368	0.001
Mix vs. Pla	3.8226	0.1011	0.001	0.4169	0.001	0.0411	0.9021	0.8650	0.001

Note: Adonis, nonparametric multivariate analysis of variance (MANOVA) with the Adonis function; ANOSIM, analysis of similarity; MRPP, multi-response permutation procedure; Con, coniferous forest; Dec, deciduous broadleaved forest; Mix, natural deciduous broadleaved and evergreen broadleaved mixed forest; Pla, plantation forest. All P-values were adjusted with the “FDR” method.

**Table 2:** Correlations between soil protistan community similarity and individual soil property based on Envfit of Principal Co-ordinates Analysis (PCoA).

Variable	PCoA1	PCoA2	r^2^	*p*
pH	0.9975	−0.0709	0.3928	0.001
SOC	−0.9965	0.0836	0.1142	0.018
TN	−0.9283	−0.3718	0.0472	0.195
TP	0.8339	0.5519	0.2767	0.001
SWC	−0.9964	−0.0847	0.3125	0.001
AN	0.2809	−0.9597	0.0086	0.758
AP	0.9260	−0.3775	0.0618	0.102
EC	0.9888	−0.1493	0.2829	0.001
C/N	−0.7462	0.6658	0.1684	0.001
C/P	−0.8050	−0.5932	0.1020	0.005
N/P	−0.8142	−0.5806	0.1014	0.005

Note: AN, available nitrogen; AP, Available phosphorus; C/N, the ratio of soil organic carbon to total nitrogen; C/P, the ratio of soil organic carbon to total phosphorus; EC, electrical conductivity; N/P, the ratio of total nitrogen to total phosphorus; SOC, soil organic carbon; SWC, soil water content; TN, total nitrogen; TP, total phosphorus.

## Data Availability

The raw sequence data from this study will be deposited in the SRA at the NCBI database after this manuscript was published.

## Abbreviations

The following abbreviations are used in this manuscript.

AN	Available nitrogen
AP	Available phosphorus
ASV	Amplicon sequence variant
C/N	Soil organic carbon/soil total nitrogen
C/P	Soil organic carbon/soil total phosphorus
EC	electrical conductivity
N/P	Soil total nitrogen/soil total phosphorus
SAR	Stramenopiles, Alveolates, Rhizaria
SOC	Soil organic carbon
SWC	Soil water content
TN	Total nitrogen
TP	Total phosphorus
PCoA	Principal coordinate analysis
